# An APSES Transcription Factor Xbp1 Is Required for Sclerotial Development, Appressoria Formation, and Pathogenicity in *Ciboria shiraiana*

**DOI:** 10.3389/fmicb.2021.739686

**Published:** 2021-09-27

**Authors:** Shuai Zhang, Panpan Zhu, Boning Cao, Shuyu Ma, Ruolan Li, Xie Wang, Aichun Zhao

**Affiliations:** ^1^State Key Laboratory of Silkworm Genome Biology, Southwest University, Chongqing, China; ^2^Key Laboratory of Biorheological Science and Technology, Ministry of Education, Chongqing University, Chongqing, China; ^3^College of Life Sciences, Xinyang Normal University, Xinyang, China; ^4^Institute of Agricultural Resources and Environment, Sichuan Academy of Agricultural Sciences, Sichuan, China

**Keywords:** APSES transcription factor, sclerotia, pathogenicity, host-induced gene silencing, *Ciboria shiraiana*

## Abstract

Sclerotinia diseases are important plant fungal diseases that, causes huge economic worldwide losses every year. *Ciboria shiraiana* is the main pathogen that results in mulberry sclerotia diseases. Sclerotia and appressoria play important roles in long-term pathogen survival and in host infection during life and disease cycles. However, the molecular mechanisms of sclerotial development and appressoria formation in *C. shiraiana* have not been well studied. Here, an Asm1p, Phd1p, Sok2p, Efg1p and StuAp (APSES)-type transcription factor in *C. shiraiana*, *CsXbp1*, involved in sclerotial development and appressoria formation was functionally characterized. Bioinformatics analyses showed that CsXbp1 contained an APSES-type DNA binding domain. The expression levels of *CsXbp1* were higher in sclerotia and during later stages of infection. Compared with wild-type strains, hyphal growth was slower, the number and weight of sclerotia were reduced significantly, and appressoria formation was obviously delayed in *CsXbp1* RNA interference (RNAi) strains. Moreover, the *CsXbp1* RNAi strains showed weakened pathogenicity owing to compound appressoria defects. Tobacco rattle virus-mediated host-induced gene silencing enabled *Nicotiana benthamiana* to increase its resistance to *C. shiraiana* by reducing the *CsXbp1* transcripts level. Thus, *CsXbp1* plays vital roles in sclerotial formation, appressoria formation, and pathogenicity in *C. shiraiana*. This study provides new insights into the infection mechanisms of *C. shiraiana* and plant resistance breeding.

## Introduction

*Ciboria shiraiana*, in the filamentous fungal genus *Ciboria*, family Sclerotiniaceae, order Helotiales of the Ascomycota (Whetzel and Wolf, 1945), is the major fungal pathogen of mulberry sclerotinia diseases, which lead to substantial reductions in production and consequent economic losses (Gray and Richard, 1987; Lv et al., 2011). Under natural conditions at a suitable temperature, the sclerotia break dormancy to generate ascospores that are released and infect the female flowers of mulberry trees. Ascospores germinate on the stigmas of female mulberry trees flowers to form bud tubes, which help ascospores invade into the stigmas and ovaries. Finally, hyphae entangle to form sclerotia in the infected mulberry fruit. The diseased fruit falls into the soil, and the sclerotia stay dormant in the soil until conditions are suitable for the next infection cycle (Lv et al., 2019).

Sclerotia play central roles in the life history of *C. shiraiana*, and they are necessary structures for the long-term pathogenicity of *C. shiraiana*. Sclerotia can germinate into fruit body to produce millions of air-borne ascospores, which are vital to the maintenance and spread of diseases in the field (Yu et al., 2012). Owing to the existence of sclerotia, sclerotinia diseases have become serious and hard to control. The compound appressorium is a multicellular infection structure that differentiates from the top of a hypha formed by pathogen. It attaches to the host surface and penetrates the plant cuticle, which helps the pathogen colonize the host plant (Uloth et al., 2016). Appressoria directly affect the pathogenic ability of the pathogen on the host plant. Therefore, it is necessary to study the growth and development of sclerotia and appressoria.

Many transcription factors have been identified as being involved in sclerotia and appressoria development in phytopathogens. For example, GATA-type zinc-finger transcription factor *SsNsd1*, Forkhead box transcription factor *SsFKH1*, MADS-Box transcription factor *BcMADS1*, and Homeobox transcription factor *MoHox2* (Kim et al., 2009; Zhang et al., 2016; Fan et al., 2017; Li et al., 2018). The APSES-type transcription factors (including Asm1, Phd1, Sok2, Efg1, and StuA) are fungi-specific transcription factors that play key roles in growth and development, secondary metabolite synthesis, and morphological transitions in fungi (Miller et al., 1992; Gimeno and Fink, 1994; Ward et al., 1995; Aramayo et al., 1996; Stoldt et al., 1997). In the phytopathogen *Magnaporthe oryzae*, APSES protein MoStu1 interacts with MoSom1 and MoCdtf1. It participates in the cyclic adenosine monophosphate (cAMP)/PKA signaling pathway to regulate hyphal growth and conidial formation, and it reduces pathogenicity by delaying appressoria formation (Nishimura et al., 2009; Yan et al., 2011). Additionally, in *M. oryzae*, the function of another APSES transcription factor has been reported. Abnormal hyphae are produced in MoSwi6 mutants owing to changes in chitin synthesis and a reduction in melanin. Moreover, the absence of MoSwi6 results in abnormal conidia, the inability of appressoria to pierce and weakened pathogenicity. MoSwi6 interacts with MoMps1 in the mitogen-activated protein kinase (MAPK) pathway and participates in downstream signal transduction (Qi et al., 2012). In addition, the StuA protein is involved in the virulence, spore to hyphal formation process and the regulation of *pks1* and *lac1* gene expression levels in the melanin-synthesis pathway of *Ustilago maydis* (Iyer et al., 2002; García-Pedrajas et al., 2010; Baeza-Montañez et al., 2015). The hyphal growth rates of *Fusarium graminearum* are inhibited in *StuA* mutants and conidia, which affect pathogenicity, by reducing the production of mycotoxin deoxynivalenol (Lysøe et al., 2011). The StuA protein in *Leptosphaeria maculans* not only participates in hyphal growth, spore formation, and pathogenicity, but it also affects effector gene expression levels (Soyer et al., 2015). The APSES protein Sok2 is the target of the cAMP/PKA pathway in *Ashbya gossypii*, and it is also an important positive regulator of sporulation (Wasserstrom et al., 2017). The APSES-like protein Vst1 has been identified in *Verticillium dahliae* and *Verticillium nonalfalfae*, and it is involved in the synthesis and pigmentation of melanin and the formation of spores (Sarmiento-Villamil et al., 2018). *FvStuA* is a regulator of sporulation, toxin synthesis, and virulence in *Fusarium verticillioides* (Rath et al., 2020).

APSES-type transcription factors play important roles in the growth, development, and virulence of phytopathogens. However, there are still limited studies that functionally characterize of APSES-type transcription factors in *C. shiraiana*. In this work, we used an RNA interference (RNAi) strategy to characterize the APSES-type transcription factor CsXbp1, which is a key component of the sclerotial development and pathogenicity in *C. shiraiana*.

## Materials and Methods

### Fungal and Plant Growth Conditions

The wild-type (WT) strain of *C. shiraiana* was cultured on potato dextrose agar (PDA) medium at 25°C. *Nicotiana benthamiana* were grown in a growth chamber at 24/18°C (day/night) with 80% relative humidity and a 16-h light/8-h-dark photoperiod.

### Bioinformatics Analysis

From the *C. shiraiana* genome, a gene encoding a fungal specific transcript factor having with an APSES-type DNA-binding domain was selected for this study. Blastp tools at the National Center for Biotechnology Information website^1^ were used to search for homologous proteins. The protein domain was predicted using the InterPro website.^2^ The Sequence alignment was performed using DNAMAN software (Lynnon BioSoft, Vaudreuil, QC, Canada) and displayed with GeneDoc software (Nicholas and Nicholas, 1997). The phylogenetic tree was constructed using MEGA7.0 software with the neighbor-joining method (Kumar et al., 2016).

### Plasmid Construction

For transcriptional activity assays, the fragments of *CsXbp1* amplified with specific primers Ta*CsXbp1*-F/R were inserted into the *SmaI* and *NotI* sites of the pGBKT7 expression vector. In the subcellular localization assays, *CsXp1* lacking the stop codon was subcloned into expression vector pYPG15-EGFP at the *BamHI* and *EcoRI* sites with primers Sub*CsXbp1*-F/R. For RNAi assays, the contain *XhoI* and *HindIII*, *KpnI*, and *BglII* target fragments of *CsXbp1* were amplified with two specific primer pairs (*CsXbp1*-F-XhoI/*CsXbp1*-R-HindIII; *CsXbp1*-F-KpnI/*CsXbp1*-R-BglII) from *C. shiraiana* cDNA and then ligated into pSilent-1 vector. pSilent-1 was then digested with *XbaI* to obtain a fragment in which the two silent segments were inserted in opposite orientations downstream of P*trpC* and a hygromycin B expression cassette. The fragment digested with *XbaI* was ligated into pCAMBIA1300 vector. For TRV-HIGS assays, a target fragment of *CsXbp1* was cloned into the pTRV2 vector using primers TRV-CsXbp1-F/R. The primers used for constructing the recombinant plasmids are listed in Supplementary Table S1. The constructed plasmids were sequenced to verify accuracy and then used for further experiments.

### Transcriptional Activation Analysis

pBD-CsXbp1 and the negative control plasmid pGBKT7 were separately transformed into yeast strain AH109 (Clontech, Tokyo, Japan). The transformants were cultured on SD/–Trp and SD/–Trp–His–Ade media at 30°C, and subsequently photographed after 3days. The α-galactosidase activity assay was performed with 5-bromo-4-chloro-3-indolyl α-D-galactopyranoside (X-α-Gal) as the substrate in accordance with the manufacturer’s instructions (Clontech, Tokyo, Japan).

### Subcellular Localization

The pYPGE15-CsXbp1-EGFP vector was transformed into *Saccharomyces cerevisiae* W303 using the polyethyleneglycol calcium method. After culturing for 18–24h, the EGFP signals in transformed yeast cells were observed under a fluorescence microscope and FV1200 confocal laser scanning microscope (Olympus, Tokyo, Japan). The localization of yeast cell nuclei was confirmed using 4',6-diamidino-2-phenylindole (DAPI) staining.

### Nucleic Acid Extraction and Quantitative Real-Time PCR

The relative expression levels of *CsXbp1* during the different developmental stages and infection process were determined. Briefly, the WT strain was cultured on PDA for 2days to collect hyphae. The sclerotia were induced by low temperature to produce apothecia, which were collected after being cultured at 25°C for 4weeks. Conidia were inoculated with hyphae and cultured in induction medium for 2weeks, washed with water, and collected by filtration through three layers of lens paper. Total RNA was extracted using TRIzol reagent according to in accordance with the manufacturer’s instructions (Invitrogen, Carlsbad, CA, United States). Total RNA (1μg) was used as the template to synthesize cDNA with the PrimeScript™ RT Reagent Kit (Perfect Real Time; Takara Tokyo, Japan). Real-time PCR was conducted using SYBRR Green I fluorescent dye detection (Takara, Tokyo, Japan). The tubulin gene served as the reference control for normalizing Ct values, and the relative expression levels of the target genes were analyzed using the relative 2^−ΔΔCt^ method (Livak and Schmittgen, 2001).

### Generation of RNAi Strain

Protoplasts of *C. shiraiana* were prepared by enzymatic hydrolysis. The RNAi vectors were introduced into protoplasts *via* polyethylene glycol-mediated transformation (Rollins, 2003). The colonies were then transferred from the regeneration medium to PDA supplemented with 60μg/ml hygromycin (Roche, Indianapolis, IN, United States), and three consecutive generation were cultivated on the selective medium. The positive transformants were verified by PCR with specific primers using genomic DNA as the templates. Three individual transformants were used for further analyses. The transcription level of the target gene in each strain was determined using quantitative real-time PCR (qRT-PCR).

### Phenotypic Characterization of RNAi Strain

For hyphal growth observation, the agar disks were inoculated on PDA plates and colony diameters were measured every 12h. Images were taken after 48h. Sclerotia were collected at 2weeks after inoculation. Then, the number and dry weight were analyzed. The appressoria were placed on a glass slide using agar plugs. The number of appressoria was measured using ImageJ software. For pathogenicity assays, healthy or wounded *N. benthamiana* leaves were inoculated using fresh PDA-colonized agar plugs of different strains and placed into an incubator for 1–2days. The lesion areas were measured using ImageJ software. All the experiments were completed with three biological independent replicates and performed three times.

### TRV-HIGS Assay

The pTRV1, pTRV2, and pTRV2: CsXbp1 vectors were transformed independently into *Agrobacterium tumefaciens* GV3101. For infiltration, transformants were cultured for 24h at 28°C and 200rpm and then collected and resuspended in infiltration buffer (10mM MgCl_2_, 10mM MES pH 5.8, and 150μM acetosyringone) to OD_600_ of 0.6–0.8 and a 1:1 mixture of pTRV1 and pTRV2 constructs. After incubation for 3h at 28°C in the dark, 4-to-6-week-old *N. benthamiana* leaves were infiltrated with bacterial suspensions using needleless syringes. The agroinfiltrated plants were then grown for 2weeks in a growth chamber at 24/18°C (day/night) with 80% relative humidity and a 16-h light/8h-dark photoperiod before *C. shiraiana* infection. Healthy *N. benthamiana* leaves were inoculated using agar plugs of different strains and placed into an incubator for 2days. The total necrotic lesion RNA extraction, cDNA synthesis, and qRT-PCR analysis were performed as mentioned before.

## Results

### *CsXbp1* Encoded an APSES-Type Protein

**Figure 1:**
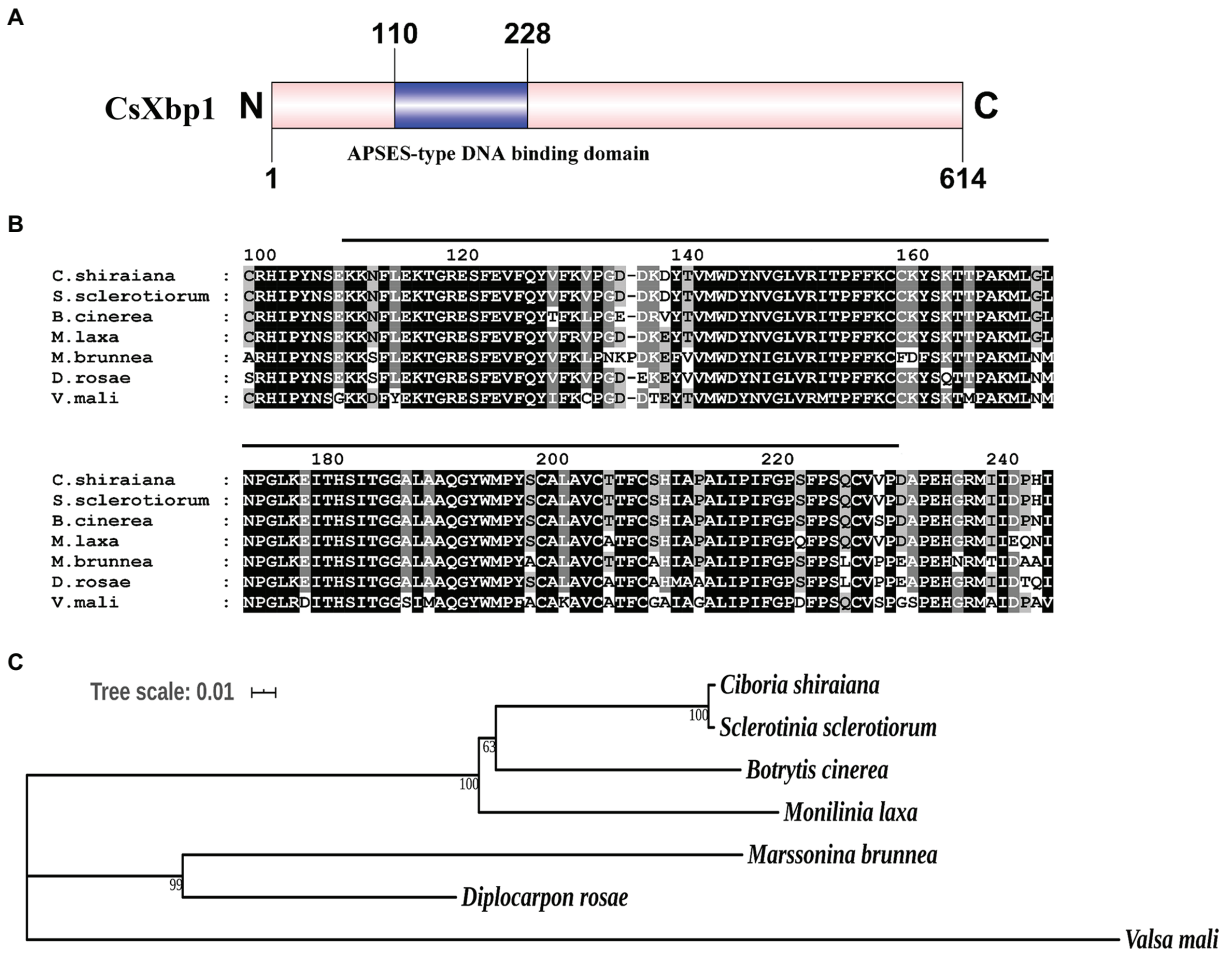
Sequence alignment and phylogenetic analysis of CsXbp1. **(A)** The predicted functional domain of CsXbp1. The full length of CsXbp1 is framed by a pink box and the APSES-type DNA binding domain is framed by a blue box. Numbers indicate the amino acid of the predicted protein. **(B)** Multiple sequence alignment of APSES-type DNA binding domain among CsXbp1 and its homologous proteins. Shading indicates sequence similarities of 100% (dark). Numbers indicate the amino acid of the predicted polypeptide. The black line indicates the APSES-type DNA binding domain region of the proteins. **(C)** Phylogenetic relationships among APSES-type DNA binding proteins. The phylogenetic tree was generated using neighbor-joining method, and bootstrap support values of 1,000 replicates are given above the branches. The scale bar corresponds to 0.01 estimated amino acid substitutions per site.

A putative fungal-specific transcription factor protein was identified in the genome of *C. shiraiana* and named CsXbp1(Cs01344) because of its homology to *Botrytis cinerea* BcXbp1. A structural analysis of CsXbp1 revealed that it contained a fungal-specific APSES-type DNA binding domain (IPR003163) that, was located from 110 to 228aa (Figure 1A). The sequence analysis of the APSES-type DNA binding domain revealed that the protein exhibited high similarities with *Sclerotinia sclerotiorum* SS1G01927 XP_001597731.1 (100% identity), *B. cinerea* B05.10 Bcxbp1 XP_024549777.1 (96.5% identity), *Monilinia laxa* EYC80003230 KAB8301349.1 (95.8% identity), *Marssonina brunnea* MBM04171 XP_007292060.1 (86.1% identity), *Diplocarpon roase* BUE80DR004326 PBP24739.1 (88.9% identity), and *Valsa mali* VM1G10544 KUI63802.1(80.6% identity; Figure 1B). The constructed phylogenetic tree revealed that CsXbp1 had close relationships with *S. sclerotiorum* and *B. cinerea* (Figure 1C).

### *CsXbp1* May Be Involved in Fungal Growth, Development, and the Infection Process

**Figure 2:**
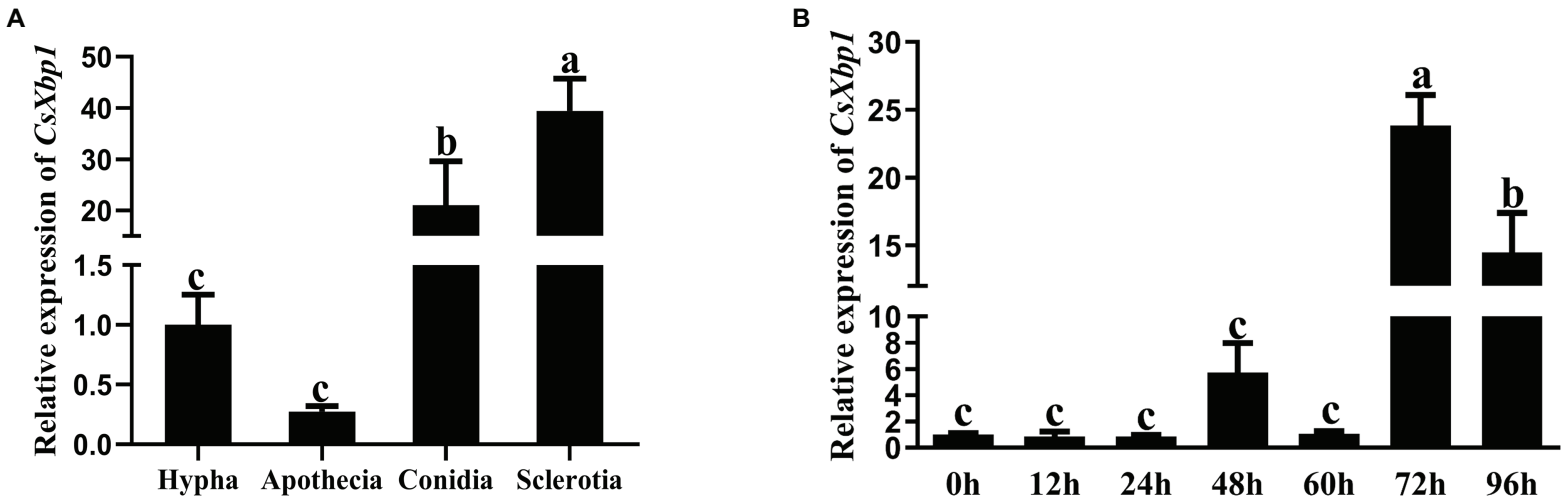
Relative expression levels of *CsXbp1* in different tissues **(A)** and during infection **(B)** as assessed by quantitative real-time PCR (qRT-PCR). The β-tubulin gene was used as the internal control to normalize the expression levels. Different letters above bars represent significant differences (*p*<0.05) as determined with using a one-way Duncan’s test. The analyses were repeated three times. Each time was investigated with three *Nicotiana benthamiana* leaves. Gene expression levels in different replicates showed similar trends.

The expression levels of *CsXbp1* during different stages of fungal development and the infection period were determined using qRT-PCR analysis. The expression level of *CsXbp1* was higher in sclerotia and conidia than in hyphae and apothecia (Figure 2A). As shown in Figure 2B, the expression level of *CsXbp1* in *C. shiraiana*-inoculated *N. benthamiana* was significantly upregulated by 23-fold at 72h post-inoculation (hpi) compared with at 0–24hpi. Thus, *CsXbp1* may participate in the regulation of sclerotial and conidial formation and plant infection.

### *CsXbp1* Localized in the Nucleus and Showed Transcriptional Activation in Yeast

In cells, the subcellular structure in which transcription factors play roles is the nucleus. To verify whether *CsXbp1* exerted its function in the nucleus, we constructed subcellular localization plasmids and transformed them into yeast. Based on an examination of fluorescence using laser microscopy and confocal fluorescence microscopy, the green fluorescent signal of recombinant plasmids overlapped the DAPI fluorescence signal (Supplementary Figures S1A,B). Thus, it confirmed that *CsXbp1* localized to the nucleus. The cells expressing empty vectors were used as negative controls.

To examine the transcriptional activity of *CsXbp1*, the recombinant plasmid pBD-*CsXbp1* and the negative control plasmid pGBKT7 were separately transformed into yeast strain AH109. Only yeast cells containing the pBD-*CsXbp1* vector can grow on the SD/–Trp–His–Ade medium (Supplementary Figure S1B), whereas yeast cells containing with negative control pGBKT7 vector cannot survive. In the assay to evaluate α-galactosidase activity, transformants containing the pBD-*CsXbp1* vector turned blue on SD/–Trp–His–Ade medium containing X-α-Gal (Supplementary Figure S1B). Thus, *CsXbp1* had transcriptional activation activity.

### Obtaining *CsXbp1* RNAi Strains

To explore the functions of *CsXbp1* in *C. shiraiana*, we used an RNA silencing strategy to obtain *CsXbp1* RNAi strains. The resulting constructs (Supplementary Figure S2A) were used to transform *C. shiraiana* protoplasts. *CsXbp1* RNAi strains were selected on PDA medium supplemented with 60μg/ml hygromycin and confirmed through the amplification of the hygromycin resistance gene (Supplementary Figure S2C). Compared with WT and empty vector (EV) controls, the *CsXbp1* expression levels were lower in the RNAi strains (Supplementary Figure S2B).

### *CsXbp1* Regulated Hyphal Growth and Sclerotial Development

**Figure 3:**
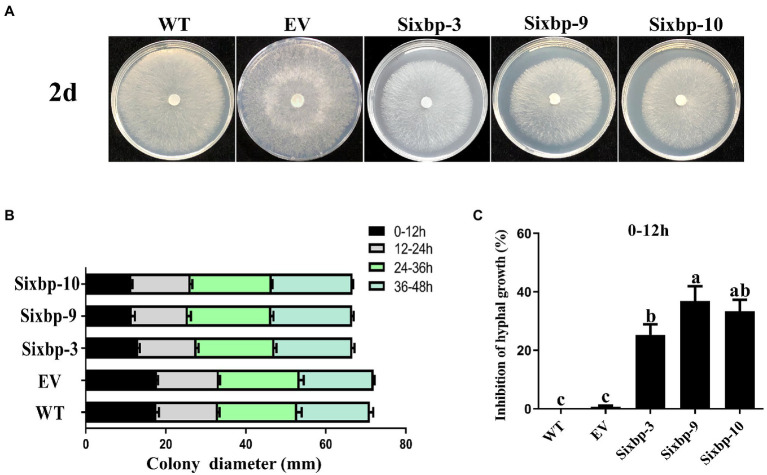
Observations of hyphal growth. **(A)** The growth of mycelia in wild-type (WT), empty vector (EV), and RNA interference (RNAi) strains. **(B)** Hyphal growth rates of the RNAi strains and the WT strains cultured on potato dextrose agar (PDA) plates. **(C)** The inhibition rates of hyphal growth. Different letters above bars represent significant differences (*p*<0.05) as determined using a one-way Duncan’s test. The experiment was repeated three times. Hyphal growth of each strain in different replicates showed similar results.

**Figure 4:**
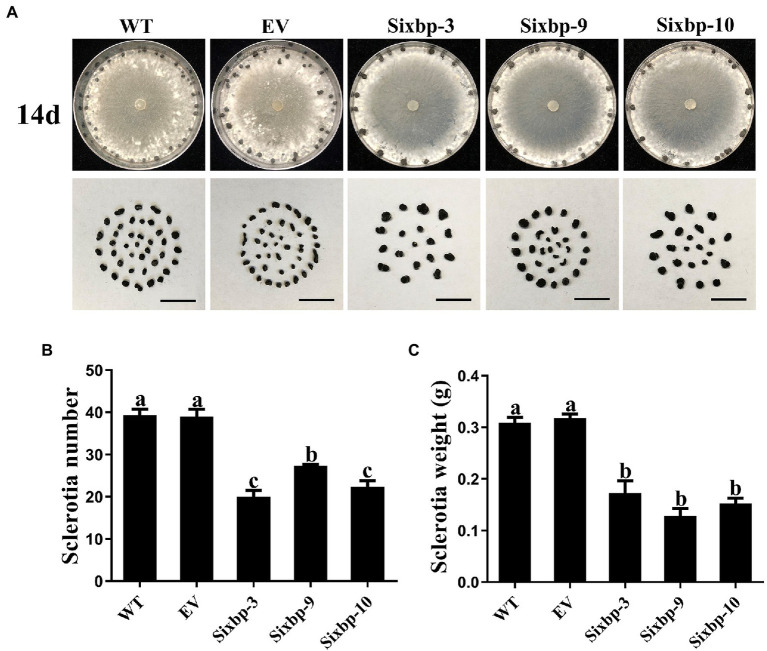
Observations of sclerotia development in WT, EV, and RNAi strains. **(A)** The sclerotial development of each strain after 14days on PDA medium. Bar, 5cm. **(B)** The number of sclerotia produced by each strain. **(C)** After 72h at 37°C, the dry weights of the sclerotia of each strain were determined. Different letters above bars represent significant differences (*p*<0.05) as determined using a one-way Duncan’s test. The experiment was repeated three times. Sclerotia development of each strain in different replicates showed similar results.

To investigate the role of *CsXbp1* in growth and development, we observed the strain phenotypes. As shown in Figures 3A,B, slower hyphal growth rates were observed in *CsXbp1* RNAi strains compared with WT and EV strains. Compared with controls, the hyphal growth inhibition rates of RNAi strains were the slowest during 0–12h, reaching 38% (Figure 3C). After these strains were cultivated for 14days on PDA medium, they all formed mature sclerotia (Figure 4A). Compared with the WT, the number of sclerotia was reduced by 30–50%, and the total dry weight decreased by 50% (Figures 4B,C). Thus, *CsXbp1* played important roles in hyphal growth and sclerotial development of *C. shiraiana*.

### *CsXbp1* Affected Compound Appressoria Formation

**Figure 5:**
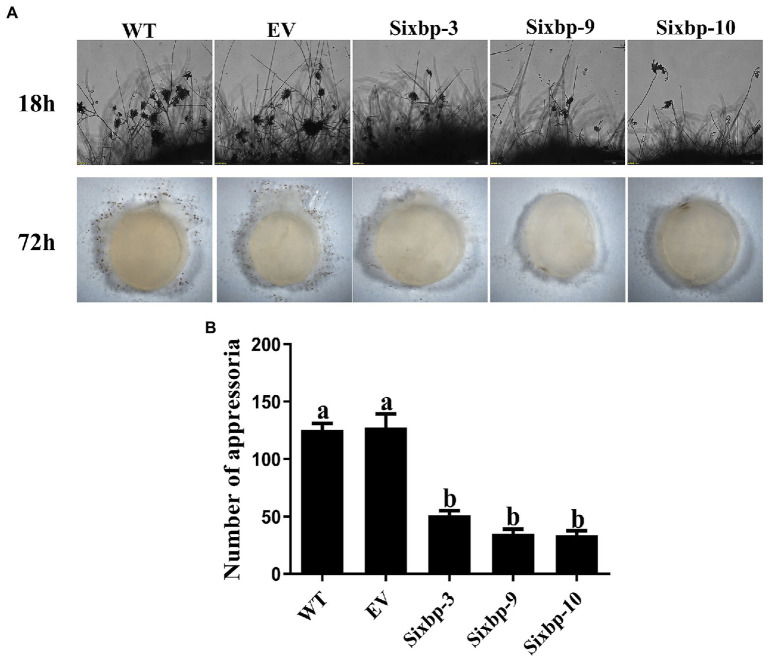
Compound appressoria formation was observed in WT, EV, and RNAi strains. **(A)** Appressoria was induced by placing the agar plugs on glass slides, and microscopic observations were conducted at 18 and 72h after induction. **(B)** The number of appressoria differentiated from each strain at 18h. Different letters above bars represent significant differences (*p*<0.05) as determined using a one-way Duncan’s test. The experiment was repeated three times. Compound appressoria formation of each strain in different replicates showed similar results.

To verify that *CsXbp1* is involved in the formation and development of compound appressoria, we conducted an appressoria induction experiment. Microscopic observations indicated that the WT and EV strains rapidly differentiated into abundant pigmented compound appressoria from vegetative hyphae at 18h. In contrast, less compound appressoria were differentiated in the *CsXbp1* RNAi strains (Figure 5A). The same results were also observed at 72h (Figure 5B), which verified that *CsXbp1* affected appressoria formation.

### *CsXbp1*-RNAi Strains Exhibited Impaired Pathogenicity

**Figure 6:**
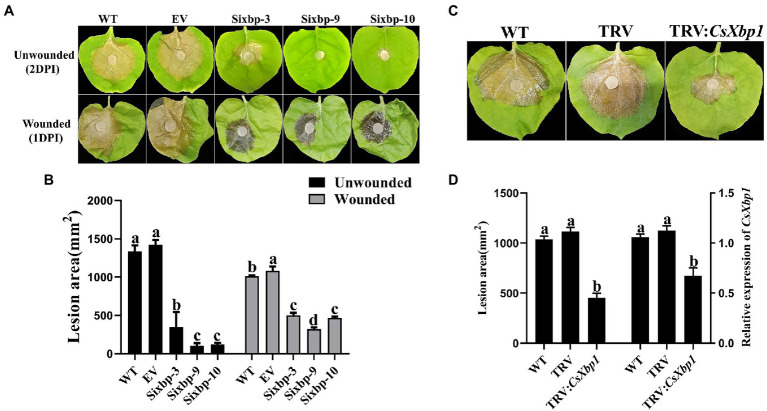
The measurement of pathogenicity in WT, EV, and RNAi strains. **(A)** *Ciboria shiraiana* infections of unwounded and wounded *N. benthamiana* leaves. **(B)** The lesion areas were measured using ImageJ software. **(C)** At 14-days-after agroinfiltration of *Agrobacterium tumefaciens* carrying the TRV-*CsXbp1* plasmid, the lesion areas were recorded. **(D)** The lesion areas were measured and evaluated the expression level of CsXbp1 in WT and agroinfiltrated plants. Different letters above bars represent significant differences (*p*<0.05) as determined using a one-way Duncan’s test. The experiment was repeated three times, and each strain was investigated with three *N. benthamiana* leaves each time.

As shown in Figure 6A, the WT and EV strains caused serious diseases on *N. benthamiana* leaves after 2days, whereas the *CsXbp1* RNAi strains caused smaller necrotic lesions. To determine whether the pathogenicity decreased owing to the defect in the appressorial production capability, the wounded leaves were inoculated. The lesions in RNAi strains were smaller than those in WT and EV strains, but larger than those on unwounded leaves (Figure 6B). Using a TRV-HIGS technology to transfer a *CsXbp1* fragment into *N. benthamiana* resulted in improved plant resistance to *C. shiraiana* by reducing the *CsXbp1* transcripts level (Figures 6C,D). Thus, our results showed that *CsXbp1* was involved in the pathogenicity of *C. shiraiana*.

## Discussion

Mulberry is widely planted in the Eurasian continent and an important tree used for the rearing of domesticated silkworm (He et al., 2013; Jiao et al., 2020). There are multiple active compounds in its fruits, such as anthocyanins, polysaccharides, and vitamins, which are beneficial to human health (Donno et al., 2015; Chang et al., 2018). The necrotrophic pathogen *C*. *shiraiana* is the main threat to mulberry fruits. A variety of methods have been used to control this pathogen, and among them, the biological control has the advantages of safety and efficiency. To develop biological control methods, the underlying mechanisms involving sclerotia and appressoria should be more comprehensively characterized. This could promote the development of some new biological control methods. The sequencing and analyses of the *C. shiraiana* genome have made it possible to identify potential target genes of biological control (Zhu et al., 2021). Here, we characterized *CsXbp1*, a fungal-specific APSES-like transcription factor in *C. shiraiana*. A series of experiments were conducted to explore its functions.

The Saccharomyces cerevisiae Xbp1 protein is a homolog of Swi4 and Mbp1, which are cell-cycle regulators (Mai and Breeden, 1997). In budding yeast, the Xbp1 protein decreases the expression levels of cyclin *CLN1-3*, *CYS3*, and *SMF2* genes by combining with their promoter regions. Furthermore, the inhibition of *CLN1* gene expression promotes sporulation (Mai and Breeden, 2000). Under nutritional deficiency conditions, Xbp1 plays a pivotal role during the transition from the cell cycle state to quiescence (Miles et al., 2013). Five APSES transcription factors (Afp1, Stu1, Mbp1, Swi6, and Xbp1) have been identified in Histoplasma capsulatum. The Xbp1 protein is specifically expressed in the yeast form but not during the mycelial phase (Longo et al., 2018). As far as we know, there is no research on the Xbp1 protein’s functions in plant pathogenic fungi. Here, we identified and functionally characterized an APSES transcription factor CsXbp1. We confirmed that CsXbp1 localized to the nucleus. As the transcriptional regulation activities of cells were mainly concentrated in the nucleus, it was reasonable that the transcription factor CsXbp1 was located in the nucleus. Further demonstrate that *CsXbp1* plays an indispensable role in different stages of fungal growth and development and in pathogenicity.

Sclerotia are asexual dormant structures that can survive for several years (Smith et al., 2015). Their exitance is one of the main reasons why pathogenic fungi are difficult to eradicate. Among them, *S. sclerotiorum* is the most notorious. The number and weight of mature sclerotia formed in the RNAi strains showed obvious downward trends, which indicated that *CsXbp1* was involved in the formation and development of sclerotia (Figures 4B,C). Thus, it was speculated that the decreased number of sclerotia would reduce the probability of long-term survival and the release of ascospores. Furthermore, the reduction in the initial infection capability and spreading area may effectively alleviate the plant infection by the pathogen. The APSES-like protein Vst1 is involved in the production of microsclerotia in *V. dahliae*, and it might be regulated by G-protein/cAMP signaling and MAPK cascades (Sarmiento-Villamil et al., 2018). The expression levels of key genes involved in sclerotia formation were reduced in the RNAi strains (Supplementary Figure S3), indicating that the *CsXbp1* may regulate sclerotial formation by participating in the cAMP-signaling pathway (Galagan et al., 2003; Rollins, 2003; Jurick Ii et al., 2004; Erental et al., 2007; Jurick and Rollins, 2007).

The necessity of *CsXbp1* for the pathogenicity of *C. shiraiana* could be observed in the inoculation assays. The pathogenicity of *C. shiraiana* to host plants was attenuated in the *CsXbp1* RNAi strains (Figure 6A). This could, in part, result from the slow growth of the *CsXbp1* RNAi strains’ hyphae, but other factors should be inolved. Oxalic acid (OA) is a significant virulence factor of pathogens (Xu et al., 2015). The pathogen secretes a large amount of OA to change the pH value in plants, aid the infection process (Bolton et al., 2006). We conducted bromophenol blue-staining experiments to determine the ability of *C. shiraiana* to secrete OA and found that there were no significant differences in the OA production between *CsXbp1* RNAi strains and the WT strain (Supplementary Figure S5), indicating that the decrease in the pathogenicity of the *CsXbp1* RNAi strains were not correlated with the OA level. Additionally, appressoria are the differentiated structures of hyphae that infect hosts to help the pathogen penetrate the first host barrier, the cell wall (Talbot, 2019). In this study, at 72h, the WT strain formed significantly more appressoria than the RNAi strains (Figures 5A,B). We then inoculated wounded leaves and found that the inoculated wounded leaves recovered some of their virulence (Figure 6A). The expression levels of key genes related to appressoria were significantly reduced in the RNAi strains (Supplementary Figure S4; Mitchell and Dean, 1995; Amselem et al., 2011; Li et al., 2012). Thus, the lack of pathogenicity in RNAi strains was mainly due to the appressorial developmental defects.

At present, the main method to prevent mulberry fruit from *C. shiraiana* infection is spraying antifungal chemicals (Lv et al., 2011). However, owing to the negative effects of antifungal chemical use, such as those affecting the environment and food safety, it is becoming increasingly important to adopt more environmentally friendly biological control methods. Molecular breeding is an important strategy to control diseases caused by this pathogen. A HIGS method was proposed as an effective strategy to improve the situation. In this method, small RNAs are produced by the host plant to target transcripts in pathogens (Koch and Wassenegger, 2021). It has been successfully applied in economically important crops, such as wheat and cotton (Cheng et al., 2015; Xu et al., 2018). We constructed the TRV-HIGS system to verify that the transfer of the *CsXbp1* gene fragment into host plants improved the resistance to *C. shiraiana* (Figure 6C). The data presented here showed that *CsXbp1* could serve as a candidate gene for mulberry disease-resistance breeding and fungicide target. In addition, the Xbp1 proteins were highly conserved among *C. shiraiana*, *S. sclerotiorum*, and *B. cinerea*. Thus, it was hypothesized that this fragment played a similar role in these two important pathogens (Dean et al., 2012; Liang and Rollins, 2018), which had broad host ranges and caused severe crop damages. Therefore, *CsXbp1* has the potential to be a target gene for controlling broad-spectrum plant diseases.

In conclusion, we used an RNA silencing strategy to explore the function of *CsXbp1*. Our results revealed sclerotial development and pathogenicity regulated by *CsXbp1*. Meanwhile, we confirmed that *CcXbp1* can be used as a potential target for disease control. Thus, HIGS may become a powerful approach to control sclerotinia diseases. The use of RNAi sequences derived from essential and fungal-specific virulence genes provides an attractive strategy and a huge pool of potential new resistance resources for the breeding of disease-resistant plants. In addition, spraying dsRNA to silence key virulence gene in pathogens might provide another effective method to control plant diseases. There should be more other functional characterization of *CsXbp1* and in-depth studies to augment the understanding of the molecular mechanisms of *CsXbp1* downstream elements to analyze the molecular pathogenic mechanism of *C. shiraiana*, which, in turn, could be used to develop novel targets for control mulberry sclerotinia disease in the future.

## Data Availability Statement

Publicly available datasets were analyzed in this study. This data can be found here: VNFM00000000.

## Author Contributions

SZ, PZ, and AZ conceived and designed the study. BC provided help in the subcellular localization assay. SM provided help in TRV-HIGS experiments. RL and XW provided the technical assistance. SZ wrote the manuscript. AZ revised the manuscript. All authors contributed to the article and approved the submitted version.

## Funding

This work was supported by grants from the National Key R&D Program of China (grant number 2019YFD1000604), China Agriculture Research System (grant number CARS-18-ZJ0201), and the National Science Foundation of Chongqing (grant number cstc2019jcyj-bsh0106). We thank reviewers for their kind suggestions.

## Conflict of Interest

The authors declare that the research was conducted in the absence of any commercial or financial relationships that could be construed as a potential conflict of interest.

## Publisher’s Note

All claims expressed in this article are solely those of the authors and do not necessarily represent those of their affiliated organizations, or those of the publisher, the editors and the reviewers. Any product that may be evaluated in this article, or claim that may be made by its manufacturer, is not guaranteed or endorsed by the publisher.

## Footnotes

^1^ http://blast.ncbi.nlm.nih.gov/Blast.cgi

^2^ http://www.ebi.ac.uk/interpro

## Supplementary Material

Supplementary Figure S1 **(A)** Subcellular localization of the CsXbp1 protein. The fluorescence channel showed the localization of GFP and CsXbp1-GFP (green). The location of the nucleus is shown by DAPI staining. Captured fluorescent images of DAPI (blue) and GFP (green) as well as a merged image, processed by ImageJ software. Bar, 10μm. **(B)** Subcellular localization of the CsXbp1 protein under confocal fluorescence microscopy. Bar, 10μm. **(C)** Transactivation analysis of the CsXbp1 protein. The transformants were selected by growth on SD–Trp medium after 3days at 30°C. Transformants were moved onto SD–Trp–His–Ade medium. The experiment was repeated three times. The experimental results are consistent.

Supplementary Figure S2 Generation of *CsXbp1* RNAi strains. **(A)** Schematic diagram of *CsXbp1* RNAi constructs. **(B)** *CsXbp1* expression levels in WT, EV, and RNAi strains. qRT-PCR was performed to generate *CsXbp1* expression profiles. The β-tubulin gene was used as the internal control to normalize the expression levels. **(C)** Amplification of the hygromycin gene from the genomes of different strains. The elongation factor gene was used as a control. Different letters above bars represent significant differences (*p*<0.05) as determined using a one-way Duncan’s test. The analyses were repeated three times. Gene expression levels in different replicates showed similar trends.

Supplementary Figure S3 Expression levels of genes involved in sclerotial formation from each strain. Different letters above bars represent significant differences (*p*<0.05) as determined using a one-way Duncan’s test. The analyses were repeated three times. Gene expression levels in different replicates showed similar trends.

Supplementary Figure S4 Expression levels of genes involved in appressorial formation from each strain. Different letters above bars represent significant differences (*p*<0.05) as determined using a one-way Duncan’s test. The analyses were repeated three times. Gene expression levels in different replicates showed similar trends.

Supplementary Figure S5 Each strain was assayed for OA production on PDA amended with bromophenol blue (50μg/ml) for 2days. The control was other RNAi strains which mycelium grows slowly but does not affect oxalic acid production. The experiment was repeated three times. OA content of each strain in different replicates showed similar results.
