# Supplementary figures and images for: An APSES Transcription Factor Xbp1 Is Required for Sclerotial Development, Appressoria Formation, and Pathogenicity in Ciboria shiraiana
Source: Front Microbiol. 2021 Sep 27;12:739686. doi: 10.3389/fmicb.2021.739686 (PMC8503677; doi:10.3389/fmicb.2021.739686)

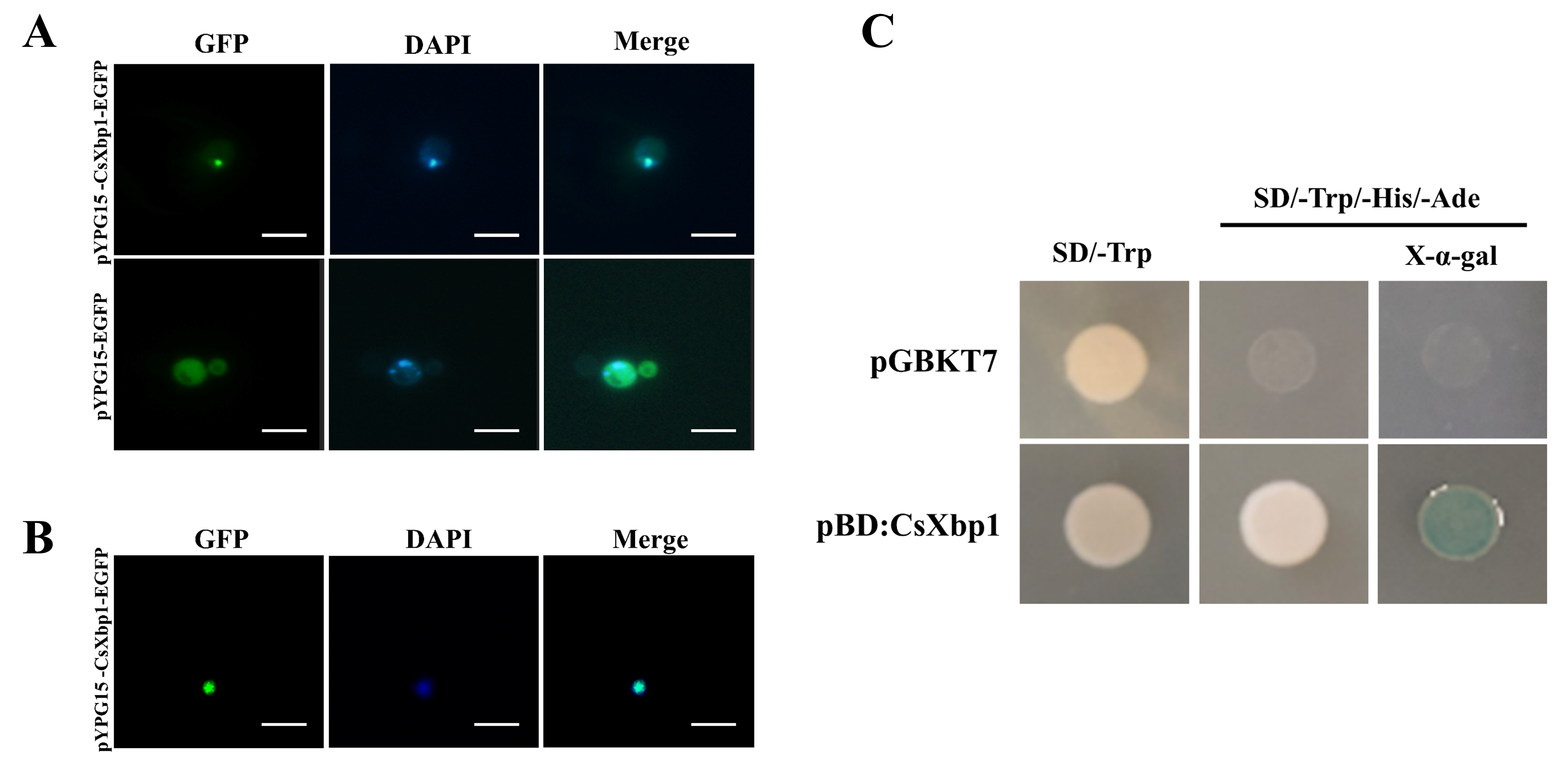

Supplement: Supplementary Figure S1 — (A) Subcellular localization of the CsXbp1 protein. The fluorescence channel showed the localization of GFP and CsXbp1-GFP (green). The location of the nucleus is shown by DAPI staining. Captured fluorescent images of DAPI (blue) and GFP (green) as well as a merged image, processed by ImageJ software. Bar, 10μm. (B) Subcellular localization of the CsXbp1 protein under confocal fluorescence microscopy. Bar, 10μm. (C) Transactivation analysis of the CsXbp1 protein. The transformants were selected by growth on SD–Trp medium after 3days at 30°C. Transformants were moved onto SD–Trp–His–Ade medium. The experiment was repeated three times. The experimental results are consistent. [file Image_1.TIF]

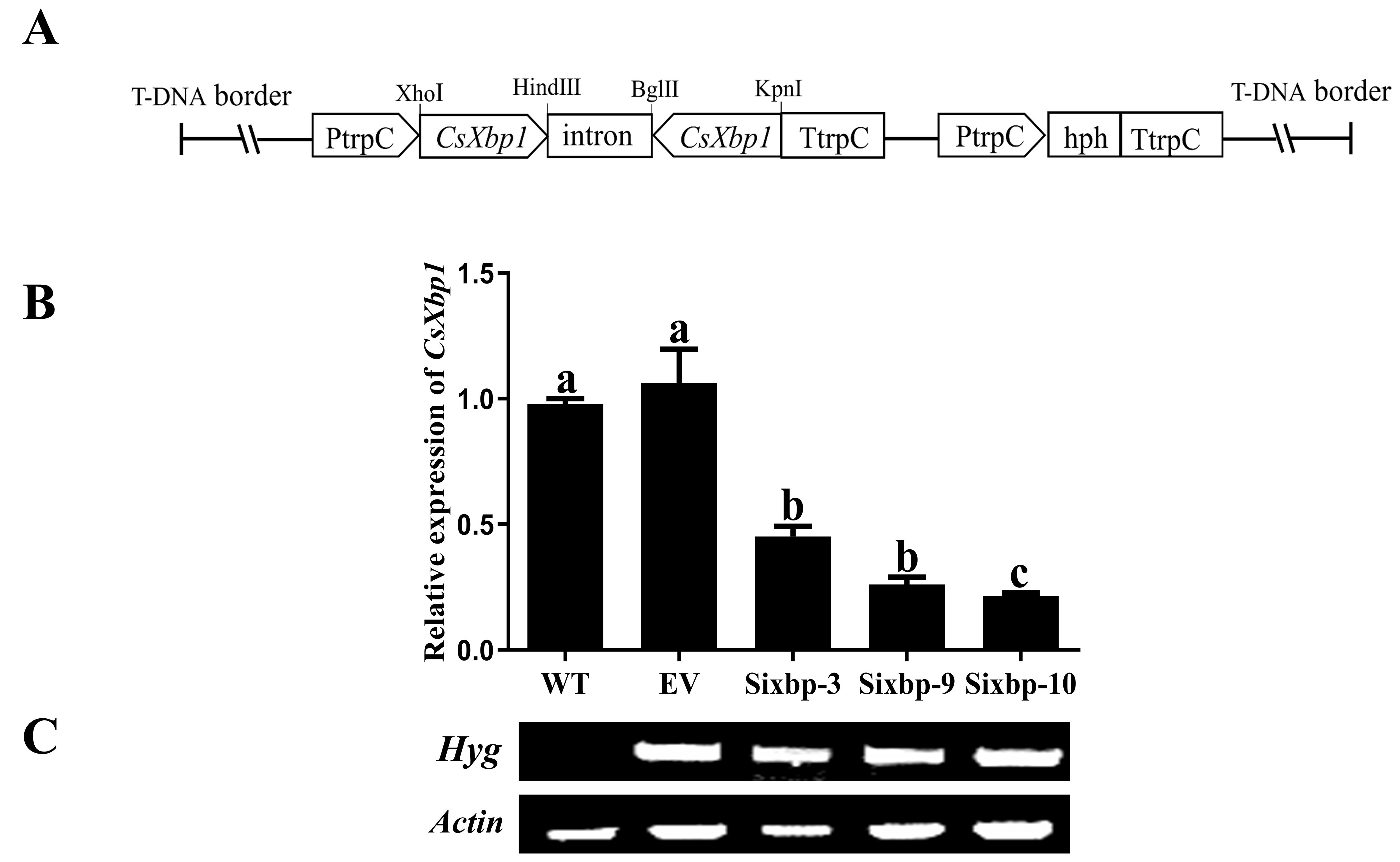

Supplement: Supplementary Figure S2 — Generation of CsXbp1 RNAi strains. (A) Schematic diagram of CsXbp1 RNAi constructs. (B) CsXbp1 expression levels in WT, EV, and RNAi strains. qRT-PCR was performed to generate CsXbp1 expression profiles. The β-tubulin gene was used as the internal control to normalize the expression levels. (C) Amplification of the hygromycin gene from the genomes of different strains. The elongation factor gene was used as a control. Different letters above bars represent significant differences (p<0.05) as determined using a one-way Duncan’s test. The analyses were repeated three times. Gene expression levels in different replicates showed similar trends. [file Image_2.TIF]

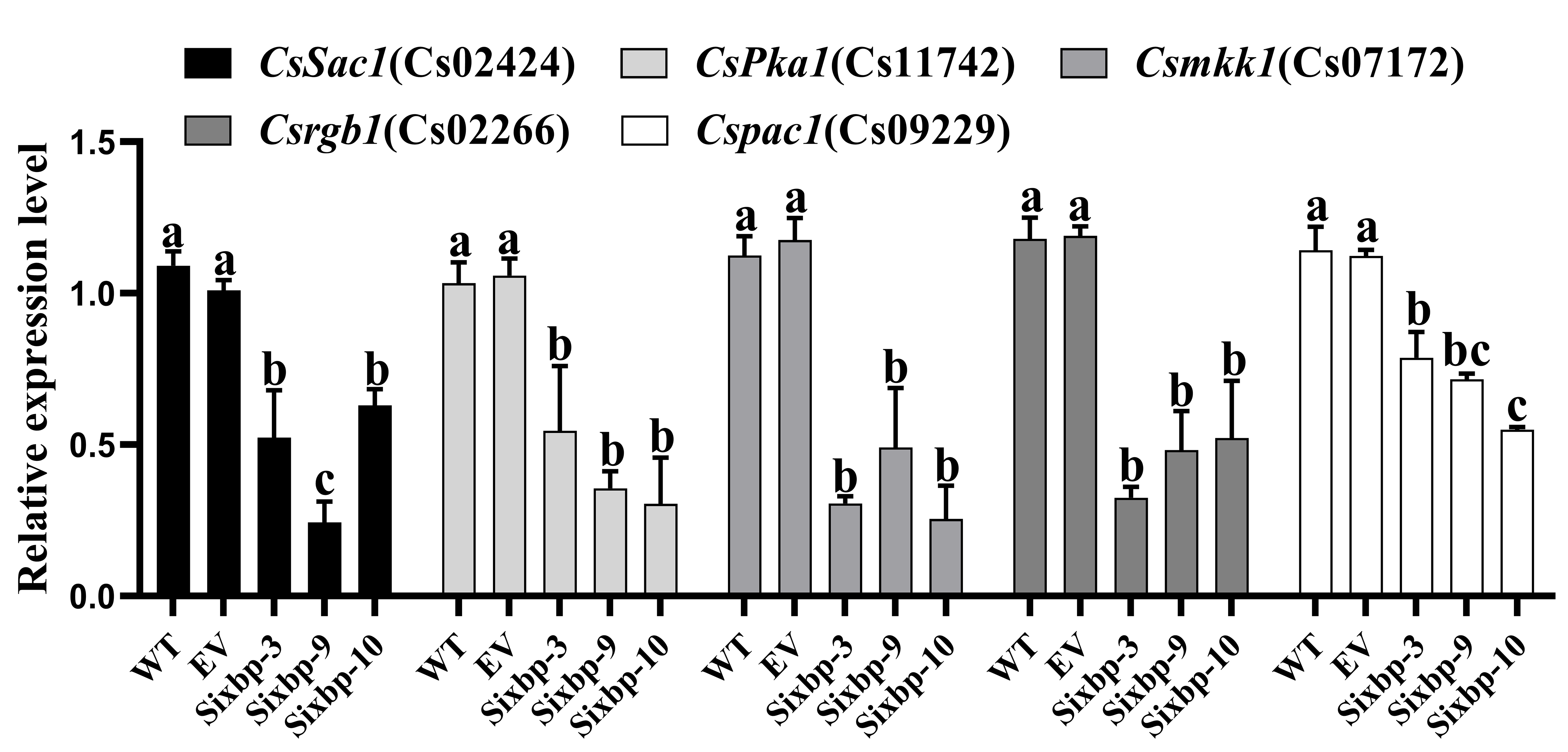

Supplement: Supplementary Figure S3 — Expression levels of genes involved in sclerotial formation from each strain. Different letters above bars represent significant differences (p<0.05) as determined using a one-way Duncan’s test. The analyses were repeated three times. Gene expression levels in different replicates showed similar trends. [file Image_3.TIF]

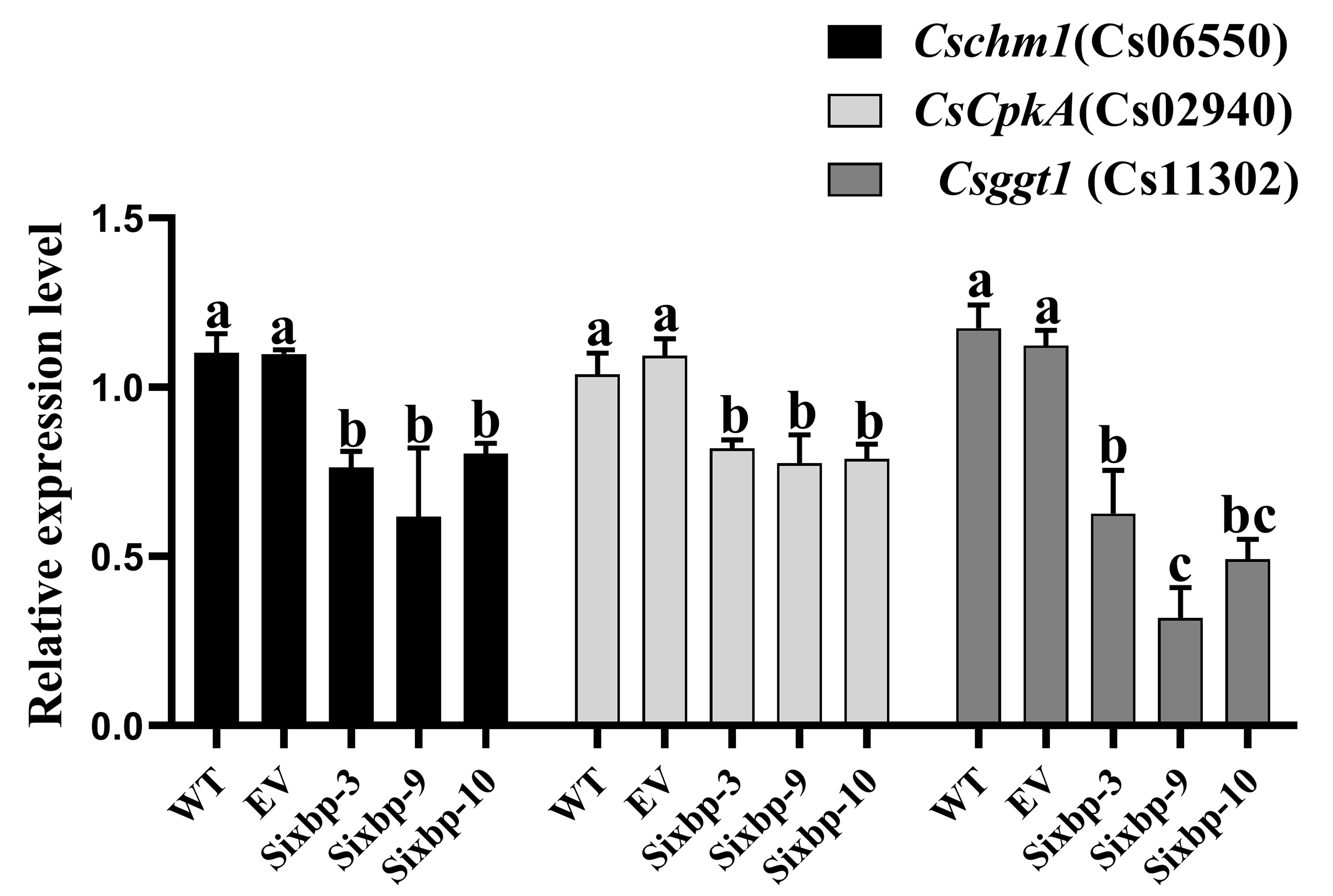

Supplement: Supplementary Figure S4 — Expression levels of genes involved in appressorial formation from each strain. Different letters above bars represent significant differences (p<0.05) as determined using a one-way Duncan’s test. The analyses were repeated three times. Gene expression levels in different replicates showed similar trends. [file Image_4.TIF]

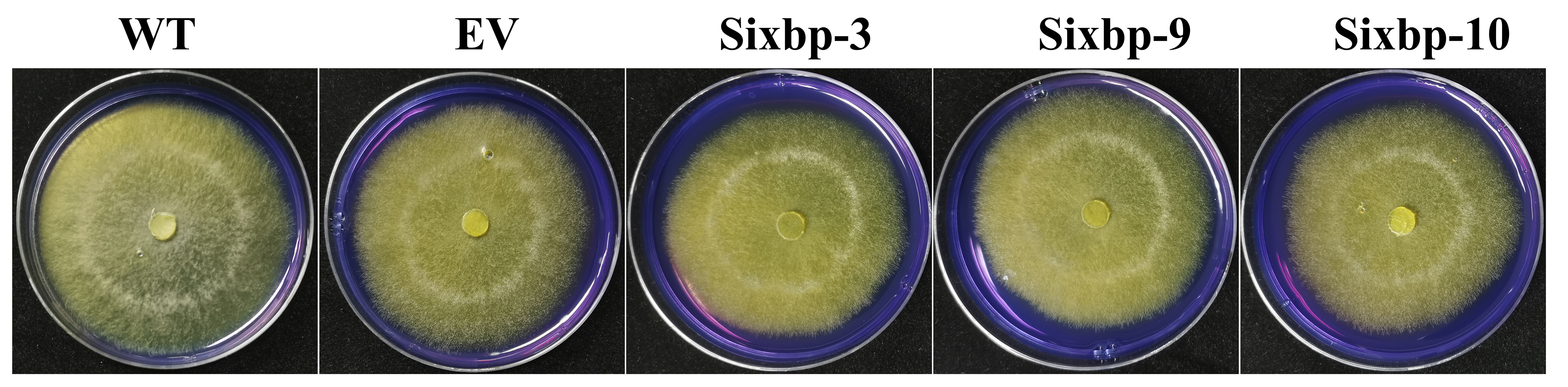

Supplement: Supplementary Figure S5 — Each strain was assayed for OA production on PDA amended with bromophenol blue (50μg/ml) for 2days. The control was other RNAi strains which mycelium grows slowly but does not affect oxalic acid production. The experiment was repeated three times. OA content of each strain in different replicates showed similar results. [file Image_5.TIF]
