# Supplementary material for: An APSES Transcription Factor Xbp1 Is Required for Sclerotial Development, Appressoria Formation, and Pathogenicity in Ciboria shiraiana
Source: Front Microbiol. 2021 Sep 27;12:739686. doi: 10.3389/fmicb.2021.739686 (PMC8503677; doi:10.3389/fmicb.2021.739686)
Supplement: Supplementary file 6 [file Table_1.DOCX]

Table S1. Sequences of the primers used in this study.

| Primer name | Primer sequence (5’-3’) | Purpose |
| --- | --- | --- |
| *CsXbp1*-F | GCATGCTGTCCGTAGCATCG | For gene cloning |
| *CsXbp1*-R | TCACATACTAAGAGCTCTCCTCC |  |
| Sub*CsXbp1*-F | ACAAGATCTCGACTCTAGAGGATCCATGCTGTCCGTAGCATCG | For subcellular localization |
| Sub*CsXbp1*-R | CTCGCCCTTGCTCACCATGAATTCCATACTAAGAGCTCTCCTC |  |
| Ta*CsXbp1*-F | TCCCCCGGGATGCTGTCCGTAGCATCG | For transcription activation |
| Ta*CsXbp1*-R | TTGCGGCCGCTCACATACTAAGAGCTCTCC |  |
| *CsXbp1*-F-XhoI | CCCTCGAGATCGTAAGTTGATGAATA | For RNAi strain constructs |
| *CsXbp1-*R-HindIII | CAAGCTTTGCCGGTGGCGAAGGGT |  |
| *CsXbp1*-F-KpnI | GGGGTACCATCGTAAGTTGATGAATA |  |
| *CsXbp1*-R-BglII | GAAGATCTTGCCGGTGGCGAAGGGT |  |
| Hyb-F | ATGAAAAAGCCTGAACTCAC |  |
| Hyb-R | CTATTCCTTTGCCCTCGGAC |  |
| EF-F | TCCTATCTCCGGTTTCAACG |  |
| EF-R | GCAAGCAATGTGAGCAGTGT |  |
| qRT*CsXbp1*-F | CCCTCGCCGTTTGCACGACT |  |
| qRT*CsXbp1*-R | CCGTGTTCTGGAGCGTCGGG |  |
| β-tubulin-F | TTGGATTTGCTCCTTTGACCAG |  |
| β-tubulin-R | AGCGGCCATCATGTTCTTAGG |  |
| TRV-*CsXbp1*-F | CGGAATTCATCGTAAGTTGATGAATA | For TRV-HIGS assay |
| TRV-*CsXbp1*-R | CGGGATCCTGCCGGTGGCGAAGGGTG |  |
| qPCR-*Csggt1*-F | CTGATTCCATTGTCGCAGTAC |  |
| qPCR-*Csggt1*-R | ACTTCAAGCGTATCGGCAT |  |
| qPCR-*Cschm1*-F | CGGCTCAGCAACAACCATCTAGG |  |
| qPCR-*Cschm1*-R | GCGGCTGGCTTGGCAGTAAG |  |
| qPCR-*CsCpkA*-F | CTGATCTGTGGCTGCTGCTGTG |  |
| qPCR-*CsCpkA*-R | CGGTGTCAAGGCTGCTGAAGG |  |
| qPCR-*CsSac1*-F | CAAGCCGAAGTCCAAGCACCTG |  |
| qPCR-*CsSac1*-R | TCCTTCTGTCTTGACCTCGTAGCC | For qRT-PCR |
| qPCR-*CsPka1*-F | TGAGCGTTCGGTTCTTGCAGATG |  |
| qPCR-*CsPka1*-R | ATCGTGCTGTGTTCTCGTCGAATC |  |
| qPCR-*Csmkk1*-F | CGAGCACATCTGTCGCTACTATGG |  |
| qPCR-*Csmkk1*-R | TGGAGATAGGTCAGACCGTTCAGG |  |
| qPCR-*Csrgb1*-F | GTTGGTGGTCAAGGTGGAAGGATG |  |
| qPCR-*Csrgb1*-R | GGTTGTTCGTTGCAGCAATCGC |  |
| qPCR-*Cspac1*-F | CAACCTGGTGCTCACTACACTCAC |  |
| qPCR-*Cspac1*-R | TTGGTGCTGAAGCGTAAGAAGTGG |  |
